# Humic Acid Extracts Leading to the Photochemical Bromination of Phenol in Aqueous Bromide Solutions: Influences of Aromatic Components, Polarity and Photochemical Activity

**DOI:** 10.3390/molecules26030608

**Published:** 2021-01-25

**Authors:** Hui Liu, Yingying Pu, Xiaojun Qiu, Zhi Li, Bing Sun, Xiaomei Zhu, Kaiying Liu

**Affiliations:** 1 College of Environmental Science and Engineering, Dalian Maritime University, Dalian 116026, China; py1120181596@dlmu.edu.cn (Y.P.); qiuxiaojun0103@dlmu.edu.cn (X.Q.); lizhi9471@dlmu.edu.cn (Z.L.); sunb88@dlmu.edu.cn (B.S.); zhuxm@dlmu.edu.cn (X.Z.); 2 School of Science, Dalian Maritime University, Dalian 116026, China; kyliuxw@dlmu.edu.cn

**Keywords:** dissolved organic matter, fractionation, photochemical activity, bromination

## Abstract

Dissolved organic matter (DOM) is considered to play an important role in the abiotic transformation of organobromine compounds in marine environment, for it produces reactive intermediates photochemically and is recognized as a significant source of reactive halogen species in seawater. However, due to the complex composition of DOM, the relationship between the natural properties of DOM and its ability to produce organobromine compounds is less understood. Here, humic acid (HA) was extracted and fractionated based on the polarity and hydrophobicity using silica gel, and the influences of different fractions (F_A_, F_B_ and F_C_) on the photochemical bromination of phenol was investigated. The structural properties of HA fractions were characterized by UV-vis absorption, Fourier transform infrared spectroscopy and fluorescence spectroscopy, and the photochemical reactivity of HA fractions was assessed by probing triplet dissolved organic matter (^3^DOM*), singlet oxygen (^1^O_2_) and hydroxyl radical (^•^OH). The influences of HA fractions on the photo-bromination of phenol were investigated in aqueous bromide solutions under simulated solar light irradiation. F_A_ and F_B_ with more aromatic and polar contents enhanced the photo-bromination of phenol more than the weaker polar and aromatic F_C_. This could be attributed to the different composition and chemical properties of the three HAs’ fractions and their production ability of ^•^OH and ^3^DOM*. Separating and investigating the components with different chemical properties in DOM is of great significance for the assessment of their environmental impacts on the geochemical cycle of organic halogen.

## 1. Introduction

Organohalogen compounds can potentially cause adverse health effects on organisms due to their high cytotoxicity and genotoxicity [1]. The vast majority of organohalogen compounds, especially organobromine compounds, have been identified to originate from natural biotic and abiotic processes in marine environments [2,3]. Apart from approximately 2000 natural organobrimine compounds that are thought to originate from biological processes, it seems that more and more attention has been paid on the abiotic formation pathways of organobromine compounds, especially through photochemical process which has a potential to produce organohalogen compounds within sunlit surface waters [4,5,6]. For example, phenol, bisphenol A and salicylic acid can be transformed into halogen-containing compounds during sunlight illumination [7,8,9]. In addition, bromophenols accounted for 93–96% of the total halogenated phenols generating from phenols in coastal seawater upon simulated sunlight irradiation [7], indicating that bromination reactions prevailed over chlorination reactions, though chloride is 760-fold more concentrated than bromide in seawater. However, many uncertainties remain about the yield of organobromine compounds in natural water, involving the bromination reagent production, the degree of the brominated reaction and its contribution to the organo-bromine pools.

Dissolved organic matter (DOM) is one of the most important natural sunlight absorbing components in aquatic environments and plays an important role in the fate of organic pollutants in natural waters, since the photosensitization of DOM yields a variety of reactive oxygen species (ROS), such as ^•^OH and ^1^O_2_ [10,11,12]. Numerous studies have demonstrated that the effects of DOM on the photodegradation of pollutants are complex, and DOM acts either as a photosensitizer or as an inhibitor depending on its different source and functional groups [13,14]. Increasing attention has been paid on the photochemical process in the presence of DOM and halides. Although halides themselves do not absorb light in the solar region, halides participate in a rich, aqueous-phase chemistry processing initiated by sunlight in the presence of DOM. For example, Parker et al. found that the photodegradation rate of microcystins was faster in saline waters relative to freshwaters in the present of DOM [15]. It was hypothesized that photochemically produced reactive halogen species (RHS), e.g., halogen radicals, might increase the indirect degradation of some pollutants. RHS, including radical RHS (i.e., X^•^, X_2_^•−^ and XY^•−^; where X=Br or I, and Y=Cl) and non-radical RHS (i.e., X_2_ and HXO, X=Cl, Br), are seawater-specific photooxidants produced by the oxidation of halides [16]. It was believed that RHS was generated from photochemical processing of DOM, where DOM-induced ROS played an important role [15,16,17]. The main reaction mechanisms are as follows: first, absorption of sunlight by DOM leads to the generation of ROS, including ^•^OH, H_2_O_2_ and DOM triplet state (^3^DOM*) [18]. Then, bromide is oxidized by the DOM-induced ROS or ^3^DOM* to form RHS [19,20]. After that, RHS reacts with other organic matters to form organobromine compounds via addition to unsaturated C−C bonds, recombination with carbon-centered radicals and/or electrophilic substitution [16,21]. However, to our knowledge, little is known on the relationship between RHS production and the natural properties of DOM.

DOM is a mixture of organic compounds with complex molecular compositions and structures, which has large diversity on physicochemical properties, including composition, polarity and aromatic contents, etc. [22]. In order to give a better insight into the structure of DOM, it is often fractionated into a series of less complex fractions. At present, various techniques have been used to separate and characterize DOM. Chromatographic techniques, including high-performance liquid chromatography (HPLC) and size exclusion chromatography, have been employed to separate and identify the functional components within DOM [23,24]. Structural properties such as polarity, hydrophobicity and aromaticity of these obtained DOM fractions are diverse, and thus they present different influences on the environmental behavior of the coexisting organic contaminants [13,23,25]. Moreover, the photo-inductive activities of DOM fractions are different from each other. Remucal et al. reported that DOM formulas with more aromatic and oxygenated components were most efficient at forming ^•^OH but less efficient at producing ^3^DOM^*^ and ^1^O_2_ [13]. Yu et al. found that the fractions of HA with different polarity that contained varied functional groups appeared to promote or inhibit the photodegradation of 2,4-D [23]. Lee et al. found that DOM with high molecular weight and hydrophobicity significantly inhibited the photolysis of target micropollutants [26]. These results indicate that the DOM separation process based on polarity and hydrophobicity is a good research method to know the relationship between DOM structure and its photochemical properties. However, little information is available for the effects of the fractions with different polarity, hydrophobicity and aromaticity on the photochemical halogenation process.

Several recent investigations have highlighted the photo-initiated halogenation of organic compounds in the presence of DOM [5,6,7,8,9,27], whereas little was done about separating and investigating the components with different chemical properties of DOM and their ability promoting RHS and even halogenation. Herein, HA was fractionated on silica gel into three subcomponents on the basis of their polarity and hydrophobicity, and then HA fractions were characterized and investigated in the process of photochemical bromination of phenol. Silica-gel chromatograph is one of the most popular fractionation methods for HA, which can separate HA into fractions according to their polarity and affinity toward silica-gel [23,25]. It is expected that more information about the effects of structures and chemical properties of DOM on RHS production and the photo-bromination process can be achieved in this study.

## 2. Results and Discussion

### 2.1. Spectroscopic Characterization of HA Fractions

#### 2.1.1. Fourier Transform Infrared Spectroscopy

**Figure 1:**
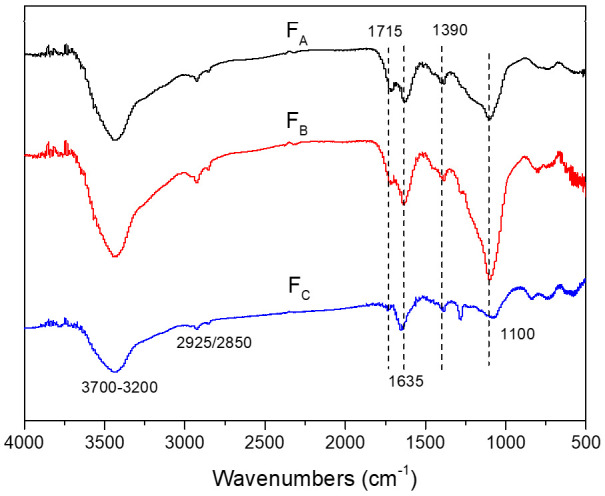
FTIR absorption spectra of three HA fractions.

Figure 1 illustrates the fourier transform infrared (FTIR) spectra of HA fractions (F_A_, F_B_ and F_C_). Generally, the spectra of HA fractions show the following bands: OH stretching vibration absorption at 3700–3200 cm^−1^, C-H stretching vibration absorption of aliphatic series at 2925/2850 cm^−1^, carbonyl C=O, aromatic C=C, hydrogen-bonded C=O, or COO asymmetrical stretch absorption around 1635/1715 cm^−1^, the symmetric COO band at 1390 cm^−1^, and C−O stretching vibration of phenol, alcohols, ethers and/or polysaccharides around 1100 cm^−1^ [28,29]. The relative peak intensities, which reflected the relative amount of each functional group, were different in the spectra of three fractions, indicating their different structural characteristics. Compared with F_C_, F_A_ and F_B_ showed relatively more intense bands at 1715, 1635, 1390 and 1100 cm^−1^, indicating that F_A_ and F_B_ contained high abundance of aromatic contents, carboxyl and phenolic functional groups.

#### 2.1.2. UV-Vis Spectroscopy

**Figure 2:**
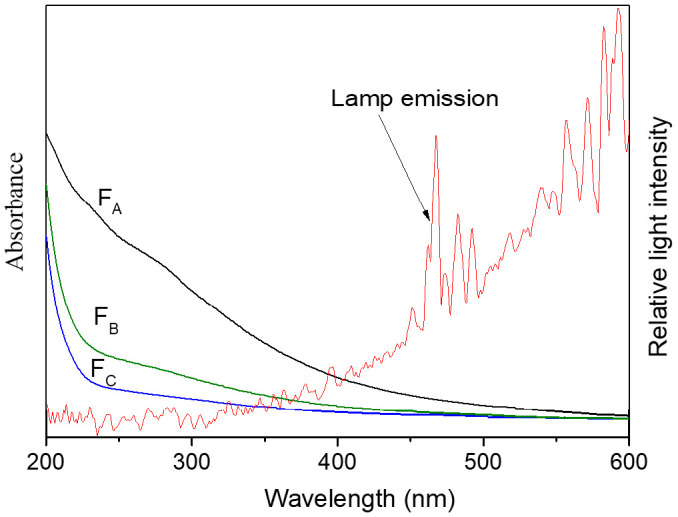
UV-visible absorption spectra of HA fractions and Xenon lamp emission.

The UV-vis absorption spectra of F_A_, F_B_ and F_C_ and the lamp emission are presented in Figure 2. The absorption of the three HA fractions varied greatly and their absorption order was F_A_> F_B_ > F_C_. Korshin et al. reported that a band corresponding to absorption around *λ* 250 nm designated π-π* transitions in the substituted benzenes or polyphenols [30]. Therefore, the specific-UV absorbance at 254 nm (SUVA_254_) was commonly correlated with the HA aromaticity [13,31]. In addition, the E_253_/E_203_ ratio was often used to characterize the type of the substituent groups on aromatic rings [32]. A low E_253_/E_203_ ratio indicates that the substituent groups on aromatic rings are mainly non-polar functional groups such as aliphatic groups, whereas a high E_253_/E_203_ ratio suggests that the main substituent groups on aromatic rings are polar functional groups such as hydroxyl, carboxyl, carbonyl and ester groups [32,33]. SUVA_254_ and E_253_/E_203_ of the three fractions are listed in Table 1. The order of SUVA_254_ is F_A_ > F_B_ > F_C_, indicating that F_A_ contains a relatively high amount of benzenoid and aromatic C=C groups compared with F_B_ and F_C_. Moreover, E_253_/E_203_ of F_A_ was apparently higher than F_B_, and F_C_ showed the lowest E_253_/E_203_ ratio. These results were well in agreement with the information provided by FTIR that F_A_ and F_B_ contained more aromatic components, and F_A_ contained more polar functional groups on its aromatic rings, such as carboxyl.

#### 2.1.3. Fluorescence Spectroscopy

**Figure 3:**
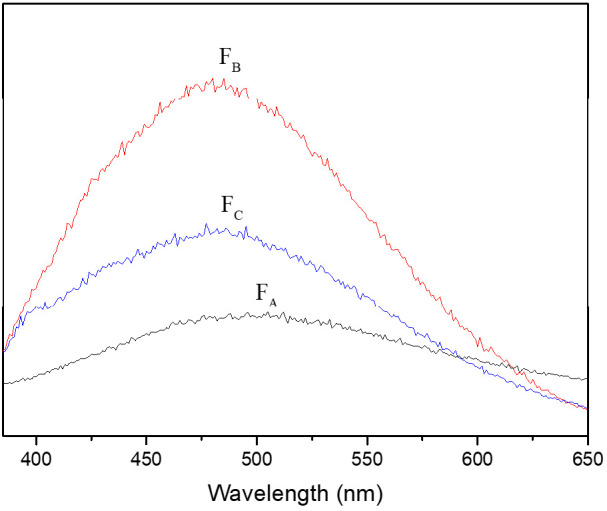
Fluorescence spectra of the HA fractions.

Fluorescence spectroscopy has been widely used to characterize DOM in water and soil [32,34,35]. The fluorescence emission spectra of the HA fractions are shown in Figure 3. The *λ*_ex_/*λ*_em_ of F_A_ was 360/502 nm with a low intensity, while the *λ*_ex_/*λ*_em_ of F_B_ and F_C_ was lower, at about 360/480 nm. The long wavelength and low intensity measured for the fluorescence peak of F_A_ indicates the presence of higher amounts of condensed aromatic rings and electron-withdrawing groups (e.g., carboxylic groups) relative to F_B_ and F_C_. On the contrary, the short wavelength and high intensity measured for F_B_ were associated with the low aromatic content and high electron-donating groups, such as the hydroxyl group [36].

Figure S1 in the Supplementary Materials shows the three-dimensional excitation-emission matrix (EEM) fluorescence spectra of HA fractions. The excitation and emission boundaries were defined into three regions based on previous literature [32,35]. Peaks at shorter excitation wavelengths (<250 nm) and shorter emission wavelengths (<350 nm) are related to simple aromatic proteins such as tyrosine (Region I, Supplementary Figure S1). Peaks at shorter excitation wavelengths (<250 nm) and longer emission wavelengths (>350 nm) are related to fulvic acid-like materials (Region II). Peaks at longer excitation wavelengths (>280 nm) and longer emission wavelengths (>380 nm) are related to humic acid-like organics (Region III). F_A_ and F_B_ were mainly composed of fulvic acid and/or humic acid, while F_C_ was mainly composed of tyrosine-like aromatic proteins. Overall, the fluorescence peaks varied depending on their polarity and aromaticity (Table 1 and Supplementary Figure S1).

Combining with UV-vis, FTIR and fluorescence data, the spectroscopic properties of HA fractions indicate that F_A_ consisted mainly of fulvic acid with plenty of aromatic components and polar functional groups on the aromatic rings, F_B_ was a mixture of fulvic acid with less aromaticity and polarity and F_C_ comprised mainly tyrosine-like aromatic proteins with low polar groups.

### 2.2. Photochemical Properties of HA Fractions

#### 2.2.1. Formation of ^3^DOM*, ^1^O_2_ and ^•^OH

The formation rate (*R*) and the quantum yields (Φ) of ^3^DOM*, ^1^O_2_ and ^•^OH of three HA fractions solutions and solution rates of light absorbance (R_abs_) are listed in Table 2. The details can be found in Text S1 and Figure S2 of the Supplementary Materials. For ^3^DOM*, *R_3DOM_* is highest in F_A_, and lowest in F_C_. However, Φ(^3^DOM*) is higher in F_B_ and F_C_ than F_A_. Φ(^3^DOM*) describes the ratio of *R_3DOM_* to light absorption. Positive correlations between absorbance and *R_3DOM_* have been observed previously [37]. The charge-transfer model of DOM photochemistry describes long-wavelength absorbance as arising from intramolecular charge transfer interactions between electron-rich donor groups (e.g., hydroxy- or methoxy-aromatic moieties) and electron-poor acceptor groups (e.g., quinones or aldehydes) that are largely derived from the partial oxidation of lignins [10,38]. These compounds should therefore be prevalent in highly aromatic DOM, i.e., F_A_. Solution rates of light absorbance significantly influence the quantum yields. Both *R_3DOM_* and R_abs_ are higher in F_A_ than F_B_ and F_C_, and R_abs_ varies by much more than *R_3DOM_*, and thus results in lower quantum yields in F_A_ (Table 2). Therefore, the trend in Φ(^3^DOM*) across the different HA portion is primarily driven by differences in R_abs_, rather than *R_3DOM_* [38].

For ^1^O_2_, measured Φ(^1^O_2_) is highest in F_C_. As shown in Table 1, F_C_ exhibited a typical EEM signature of aromatic protein contents, while the typical components of F_A_ and F_B_ were humic acid and fulvic acid. It has been reported that the protein-like components had higher ^1^O_2_ quantum yields than that of fulvic acid and humic acid [38,39]. Therefore, F_C_ was highly efficient at forming ^1^O_2_, which was in agreement with previous observations that found that more saturated formulas that are common in microbially derived DOM are strongly correlated with the formation of ^1^O_2_ [39].

^3^DOM* is a precursor for ^1^O_2_ and the yield for this process is quite high [40,41], thus the order of Φ(^1^O_2_) and Φ(^3^DOM*) was similar to F_C_ > F_A_ and F_B_. In addition, Φ(^1^O_2_) (range = (2.69 ~ 4.31) × 10^−3^) was almost greater than Φ(^3^DOM*) (range = (1.38 ~ 2.67) × 10^−3^) in all fractions. It should be noticed that ^1^O_2_ and 2,4,6-trimethylphenol (TMP) likely probe different pools of ^3^DOM*, which may be attributable to the different ^3^DOM* populations capable of reaction with O_2_ and TMP, respectively. Almost all triplets that react by energy transfer are theorized to be able to be quenched by O_2_, since the singlet energy of O_2_ (94 kJ mol^−1^) is much lower than the reported average triplet energy of DOM (~175 kJ mol^−1^) [10,40]. In contrast, TMP has a one-electron oxidation potential of 1.22 V, and it reacts by electron transfer with ^3^DOM* that has an excited state reduction potential greater than that value [40]. Thus, only triplets with reduction potentials sufficient to oxidize TMP could be detected in ^3^DOM* quantification experiments.

For ^•^OH, the *R*_•OH_ order for HA fractions is F_A_ > F_B_ > F_C_, similar to ^3^DOM*, whereas, after normalization to the rates of light absorbance, Φ(^•^OH) order was F_B_ > F_C_ > F_A_ (Table 2). In fact, DOM is considered as the main source of ^•^OH in surface waters [42,43]. The main formation process of ^•^OH involves the formation of H_2_O_2_ by irradiated DOM, followed by the generation of ^•^OH via direct photolysis or photo-Fenton processes. Another ^•^OH formation is considered via oxidation water by excited DOM. However, conflicting evidence exists about the relationships between the formation of ^3^DOM* and ^•^OH. In several studies, DOM samples with the highest Φ(^3^DOM*) have the highest Φ(^•^OH), implying that ^3^DOM* plays the primary role in ^•^OH formation [11,43,44]. In contrast, other studies concluded that DOM that was highly efficient at producing ^3^DOM* was inefficient at forming ^•^OH, suggesting that ^•^OH was generated through a non-^3^DOM* species precursor [13,45]. Overall, the photoproduction of ^•^OH is complicated, and the relationship between the composition and properties of DOM and ^•^OH formation needs further investigation.

#### 2.2.2. Photochemical Bromination of Phenol in the Presence of HA Fractions

**Figure 4:**
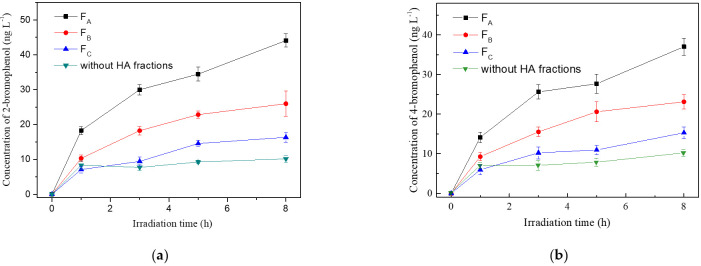
Effects of different HA fractions on the photochemical generation of 2-bromophenol (**a**) and 4-bromophenol (**b**). [phenol]_0_ = 2 mg L^−1^, [Br^−^] = 8 mmol L^−1^ and [HA] = 6.3 mgC L^−1^.

The generation of bromophenols in bromide solutions containing different HA fractions under simulated sunlight irradiation are shown in Figure 4. The concentration of bromide, [Br^−^], in seawater is at an average concentration of 0.8 mmol L^−1^, while it is enriched in the sea-spray aerosols due to the evaporation of water and can reach dozens of mmol L^−1^ [46]. Considering the wide [Br^−^] range in seawater and aerosol, the bromination of phenol was investigated in the presence of 8 mmol L^−1^ Br^−^ in this study. The initial phenol concentration is 2 mg L^−1^, and the concentration of HA fractions is 6.3 mgC L^−1^. Phenol was irradiated in the reaction solutions and formed two brominated derivatives, 2-bromophenol and 4-bromophenol. The fractions with high aromatic contents and polarity, F_A_ and F_B_, enhanced phenol bromination obviously, and the promotion effects of each fraction on the photo-bromination of phenol varied in the order of F_A_ > F_B_ > F_C_.

As it has been reported previously, phenolic compounds and natural organic substances could be brominated in the sunlit saline solutions or seawaters, where reactive bromine species (RBS) play an important role [5,9,16,19]. RBS includes bromide radicals (Br^•^/Br_2_^•−^) and non-radical RBS (HOBr and Br_2_). The bromine radicals could be generated through oxidation of bromides by ^•^OH (Equations (1)–(3)) [17,19], or by ^3^DOM*, such as excited triplet state ^3^HA* (Equation (4)) [16], whereas the non-radical RBS could be generated via the recombination of radical intermediates (Equations (5)–(8)). Therefore, the influence of HA fractions on the bromination of phenol should be focused on the capacity of HA fractions to produce bromine radicals.

^•^OH+ Br^−^ → HBrO^•−^ (1)

HBrO^•−^ + H^+^ → Br^•^ + H_2_O (2)

Br^•^ + Br^−^ → Br_2_^•−^ (3)

^3^HA^*^ + Br^−^ → HA^•−^ + Br^•^ (4)

Br^•^ + Br^•^ → Br_2_ (5)

Br_2_^•−^ + Br_2_^•−^ → Br_2_ + 2 Br^−^ (6)

Br^•^ + ^•^OH → HBrO (7)

Br_2_^•−^ + ^•^OH → HBrO + Br^−^ (8)

**Figure 5:**
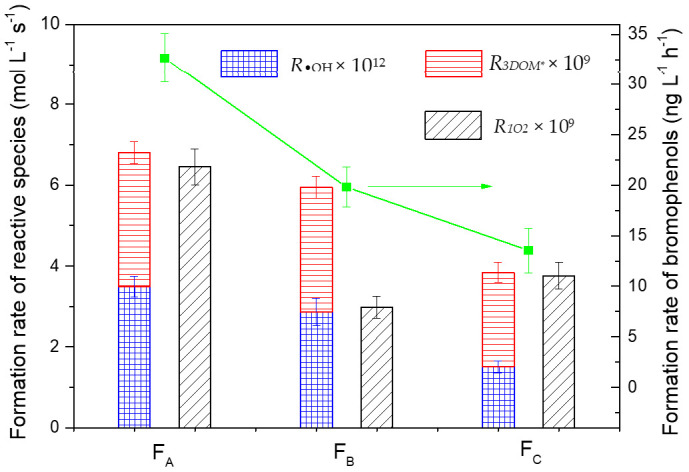
Relationship between the formation rate of reactive species and the total formation rate of bromophenols (2-bromophenol + 4 bromophenol) among three HA fractions. Reaction conditions: [Br^−^] = 8 mmol L^−1^, formation rates of bromophenols are the values calculated at t = 1 h, and the error bars represent one standard deviation.

One important thing we wanted to explore was the relationship between the physico-chemical properties of HA fractions and the photo-bromination reactions. Figure 5 shows that the formation rate of bromophenols was in the order F_A_ > F_B_> F_C_, which was consistent with the sum of the formation rates (*R*) of ^3^DOM* and ^•^OH for the three HA fractions. There is no evidence for the generation of RBS from ^1^O_2_ yet, and also there was no correlation between formation rate of bromophenols and ^1^O_2_. These results indicated that the higher polar and aromatic fraction (F_A_) with composition of fulvic acid was prone to produce bromophenols, most probably due to ^3^DOM* and ^•^OH generated by F_A_.

Oxidation of bromide by ^•^OH (E = 2.8 V_NHE_), forming Br radical (E = 1.7–2.0 V_NHE_), is non-selective and has long been recognized as a source of halogen radicals in seawater [16,20]. The production of ^•^OH from DOM could occur via photo-generated H_2_O_2_ from DOM, or oxidation of water and/or OH^−^ by photochemically excited DOM. However, the relationship between composition of DOM and ^•^OH formation has not been fully understood yet. Remucal et al. found that DOM formulas with more aromatic components are most efficient at forming ^•^OH [13]. This finding could support our results that F_A_ accelerated bromination obviously, since F_A_ contains higher aromatic contents (Figure 1) and can form ^•^OH at a higher rate (Table 2), and consequently can generate more RBS. Meanwhile, the formation rate of bromophenols became slow during irradiation, which may be related to the structural change of HA fractions. Various studies have shown that aromatic components of DOM are susceptible to photooxidation and destroyed during irradiation [10,47,48]. The loss of aromatic components might decrease the generation of RBS, and thus slow the bromination rate.

Another pathway for RBS formation is direct oxidation of bromides by ^3^DOM*. Parker et al. demonstrated that RBS formation via ^3^DOM* oxidation of halides may be a significant RBS generation pathway in coastal seawater [15,17]. As we know, properties of ^3^DOM^*^ are clearly different for various structural components [49], and whether ^3^DOM^*^ can oxidize bromide ions to radicals depends on its standard reduction potential. Generally, the estimated ^3^DOM* reduction potentials (E(^3^DOM*/DOM^•^^−^) = 1.3−1.9 V_NHE_) [40] fall within the range of reduction potentials to oxidize halides (E(Br_2_^•^^−^/Br^−^) = 1.7 V_NHE_) [16]. Moreover, DOM exists as super-molecular aggregates and colloids, where the reduction potential of bromide radical should be lower than in aqueous solutions. The reason is that the estimated reduction potential of bromide radical is about 0.4–0.5 V_NHE_ lower in polar organic solvents, and the electric field in the vicinity of the chromophoric site of DOM may be somewhere in between water and polar organic solvents [40]. Therefore, the oxidation of bromide in the DOM microenvironment should be easier than in water phase.

The aromaticity and polarity of DOM fractions could influence the property of DOM microenvironment. Although the relationship between DOM composition and the oxidizing ability of ^3^DOM* is not known yet, it has been reported that some DOM proxies, such as anthraquinone-2-sulphonate, possess a powerful triplet oxidant to be 2.28 V_NHE_ [50]. Thus, it can be supposed that aromatic fractions (F_A_ and F_B_) may have a relatively stronger oxidizing ability of ^3^DOM* due to the possible existence of aromatic ketones structures. In addition, the π-π complexes of DOM and phenolic compounds were supposed to impact their photochemical reaction [26]. It has been demonstrated that π-π electron donor-acceptor interactions played an important role between π-donor aromatic compounds and π-acceptor DOM, since DOM contains lots of potentially strong π-acceptor groups, such as quinones and aromatic rings substituted with electro-withdrawing groups such as carbonyl and carboxyl [51]. FTIR spectra demonstrated that F_A_ possessed more aromatic C=C contents, and UV-vis absorbance (E_253_/E_203_) demonstrated that F_A_ contained more polar groups, such as carboxyl, on aromatic rings. Therefore, the higher aromatic and stronger polar F_A_ was a stronger π-acceptor than F_B_ and F_C_. It is supposed that F_A_ and phenol formed stronger complexes than F_B_ and F_C_, which provided a better microenvironment for phenol reaction with reactive species (including both ROS and RBS) to generate bromophenols.

## 3. Materials and Methods

### 3.1. Standards and Reagents

Phenol, 2-bromophenol, 4-bromophenol, furfuryl alcohol (FFA), terephthalate (TPA), 2,4,6-trimethylphenol (TMP), *p*-nitroanisole (PNA) and pyridine were purchased from Sigma-Aldrich, and 2-hydroxy-5-chlorobiphenyl was purchased from AccuStandard. All chemicals were with the purity > 98% and used as received. HA was purchased from MP Biomedical, Inc (Eschwege, Germany). Deionized water (18 MΩ cm) was obtained from a Milli-Q system and used in all experiments.

### 3.2. Fractionation Procedure of HA

The fractionation procedure of HA was described briefly as follows: stock solution of HA was loaded onto a silica gel (60–80 mesh) chromatogram column (Ø30 × 300 mm). A mixture of ethanol and water was selected as the mobile phase and the flow rate was about 1.0 mL min^−1^. Column effluents were collected as 150 mL aliquots using different volume proportions of ethanol and water in the sequence of the ratio of ethanol:water at 3:7, 4:6 and 5:5, and then three HA fractions, F_A_, F_B_ and F_C_, were obtained. Then, the pH of HA solution was adjusted to 1.0 by 6 mol L^−1^ HCl for precipitation, and the precipitates were filtrated and dried at 60 °C for 6 h. The obtained solids were finally dissolved in alkaline solutions (pH 8.5) for total organic carbon (TOC) measurement and used for further experiments.

### 3.3. Characterization of HA Fractions

The functional groups of HA fractions were analyzed by Fourier transform infrared spectroscopy (FTIR, Nicolet iS 5, Thermo Fisher Scientific, Madison, USA) in the wave number range of 4000 to 400 cm^−1^. The light absorption properties of HA fractions were characterized using UV-vis spectrophotometry (Hitachi UH5300, Ibaraki, Japan). Fluorescence spectra of the HA fractions were obtained using a Hitachi F-4500 fluorescence spectrophotometer (Japan). The emission spectrum between 380 and 650 nm with excitation at 360 nm was recorded, and excitation-emission matrix (EEM) spectra were obtained by continuous scanning of the emission (Em) wavelength from 220 to 600 nm by increasing the excitation (Ex) wavelength from 200 to 450 nm. TOC was measured using a TOC analyzer (LiquiTOCII, Elementar Analysensysteme GmbH, Langenselbold, Germany).

### 3.4. Photochemical Experiments

The photochemical experiments were conducted in cylindrical quartz tubes, and the simulated sunlight source (Phchem III, Beijing Newbit Technology Co., Ltd., Beijing, China) contains a 500 W xenon arc lamp and filters to cut off the light with a wavelength below 290 nm. The lamp emission spectrum (shown in Figure 2) was similar to the wave band 290–450 nm of sunlight that plays a main role in photochemical reaction. Photo-productions of ^3^DOM*, ^1^O_2_ and ^•^OH irradiated with a Xenon lamp were quantified using TMP, FFA and TPA as chemical probe molecules, respectively. Experiments were performed in triplicate alongside a *p*-nitroanisole/pyridine actinometer, which was used to quantify light intensity [52]. Experimental details and the determination of photo-formation rates of ^3^DOM*, ^1^O_2_ and ^•^OH are provided in Text S1 of the Supplementary Materials.

### 3.5. Chemical Analysis

The concentrations of bromophenols were analyzed by gas chromatography mass spectrometry (GC-MS, Aglient 7890B/5977C, Agilent Technologies, Santa Clara, CA, USA) after extraction from aqueous solutions using dichloromethane. The concentrations of TMP and FFA were measured using high-performance liquid chromatography (HPLC) and the concentration of HTPA was detected using a fluorescence spectrophotometer. Details are in Text S2 of the Supplementary Materials.

## 4. Conclusions

HA was fractionated into three fractions based on the polarity and hydrophobicity using silica gel. F_A_ consisted of mainly fulvic acid with plenty of aromatic contents and polar functional groups on the aromatic rings, F_B_ was a mixture of fulvic acid with less aromatic contents and polarity and F_C_ comprised mainly tyrosine-like aromatic proteins with low polar groups. The promotion order of HA fractions on the photobromination of phenol was F_A_ > F_B_ > F_C_, which was consistent with the order of formation rates of ^3^DOM* and ^•^OH for the different HA fractions. The higher aromatic and polar fraction accelerated phenol bromination due to its production of ^•^OH and ^3^DOM* and the chemical property of the DOM microenvironment. The present work is an attempt to gain new insight into the separation, recognition and assessment of DOM environmental impacts on photochemical formation of organobromine compounds in marine environment.

## Figures and Tables

**Table 1:** Spectroscopic properties of HA fractions (F_A_, F_B_ and F_C_).

Sample	SUVA ^a^ (L mg^−1^m^−1^)	E_253_/E_203_^b^	EEM ^c^ Spectra	
			Peak	Property
F_A_	4.73	0.65	II, III	Fulvic and humic-like
F_B_	1.65	0.32	I, II	Fulvic and protein-like
F_C_	0.87	0.22	I	Protein-like

^a^ Specific UV absorbance at 254 nm (A254/organic carbon content). ^b^ Ratio of absorbance at 253 and 203 nm. ^c^ Three-dimensional excitation-emission matrix fluorescence.

**Table 2:** Quantum Yields (Φ) and formation rates (*R*) of ^3^DOM*, ^1^O_2_ and ^•^OH, and R_abs_ in solutions containing HA fractions (6.3 mgC L^−1^). The values were calculated based on Equations (S1)–(S8) of the Supplementary Materials.

	Φ (^3^DOM*) × 10^3^	R_3DOM*_ × 10^9^ mol L^−^^1^ s^−1^	Φ (^1^O_2_) × 10^3^	*R_1O2_* × 10^9^ mol L^−^^1^ s^−1^	Φ (^•^OH) × 10^6^	*R*_•OH_ × 10^12^ mol L^−^^1^ s^−1^	R_abs_ × 10^9^ Es L^−3^ s^−1^
F_A_	1.38 ± 0.11	3.31 ± 0.27	2.69 ± 0.19	6.45± 0.44	1.49 ± 0.10	3.49± 0.26	2.19
F_B_	2.47 ± 0.22	3.09 ± 0.27	2.57 ± 0.23	2.98 ± 0.27	2.37 ± 0.28	2.86 ± 0.34	1.23
F_C_	2.67 ± 0.29	2.33 ± 0.25	4.31 ± 0.37	3.76 ± 0.33	1.73 ± 0.17	1.51 ± 0.15	0.89

## Data Availability

The data presented in this study are available in the article and supplementary material.

## Supplementary Materials

The following are available online. Text S1: Detailed calculation of formation rate (*R*) and the quantum yields (Φ) of ^3^DOM*, ^1^O_2_ and ^•^OH and solution rates of light absorbance (R_abs_) of the three HA fractions, Text S2: Analysis of bromophenol products, TMP, FFA, HTPA and PNA, Figure S1: EEM fluorescence spectrum of F_A_, F_B_, and F_C_., Figure S2: The curve of each probe molecule and PNA over time under simulated sunlight.
