# Supplementary material for: Humic Acid Extracts Leading to the Photochemical Bromination of Phenol in Aqueous Bromide Solutions: Influences of Aromatic Components, Polarity and Photochemical Activity
Source: Molecules. 2021 Jan 25;26(3):608. doi: 10.3390/molecules26030608 (PMC7926322; doi:10.3390/molecules26030608)
Supplement: Supplementary file 1 [file molecules-26-00608-s002.pdf]

Supplementary Material

Humic Acid Extracts Leading to the Photochemical Bromination of Phenol in Aqueous Bromide Solutions: Influences of Aromatic Components, Polarity and Photochemical Activity

Hui Liu [1,*], Yingying Pu [1], Xiaojun Qiu [1], Zhi Li [1], Bing Sun [1], Xiaomei Zhu [1] and Kaiying Liu [2]

1 College of Environmental Science and Engineering, Dalian Maritime University, Dalian 116026, China; py1120181596@dlmu.edu.cn (Y.P.); qiuxiaojun0103@dlmu.edu.cn (X.Q.); lizhi9471@dlmu.edu.cn (Z.L.); sunb88@dlmu.edu.cn (B.S.); zhuxm@dlmu.edu.cn (X.Z.)

2 School of Science, Dalian Maritime University, Dalian 116026, China; kyliuxw@dlmu.edu.cn

* Correspondence: liuhui@dlmu.edu.cn

Text S1

Detailed calculation of formation rate (R) and the quantum yields (Φ) of $^3DOM^*$, $^1O_2$ and $^•OH$ and solution rates of light absorbance (R_{abs}) of the three HA fractions.

Probe compound stock solutions were prepared in Milli-Q water and spiked into water samples containing HA fractions. 2,4,6-Trimethylphenol (TMP), furfuryl alcohol (FFA), and terephthalic acid (TPA) were used to measure $^3DOM^*$, $^1O_2$, and $^•OH$, respectively. In the measurements of $^3DOM^*$ and $^1O_2$, methanol (0.1 M) was added to quench the photogeneration of $^•OH$. In the determination of $^3DOM^*$, an initial concentration of 1 mM TMP was added into the sample. The solutions were sparged with N_2 to remove dissolved oxygen. The degradation rate constant of TMP (k_{TMP}, s^{-1}) was fitted by pseudo-first order kinetics. The initial transformation rate (M s^{-1}) of TMP was defined as $R_{TMP} = k_{TMP}\,[TMP]_0$. The formation rate of $^3DOM^*$ ($R_{^3DOM^*}$, M s^{-1}) could be obtained from Equation S1 [1,2].

$$R_{TMP} = R_{^3DOM^*}\frac{k_{TMP,^3DOM^*}[TMP]_0}{k_{TMP,^3DOM^*}[TMP]_0 + k_d} \quad \text{(S1)}$$

where $k_{TMP,^3DOM^*}$ is the second-order rate constant between $^3DOM^*$ and TMP (~ 3.0×10^9 $M^{-1}\,s^{-1}$); k_d is the physical quenching constant of $^3DOM^*$ (~ 5.0×10^4 s^{-1}) [2]. Addition of 1 mM of TMP in this study yielded a scavenging rate of approximately 3×10^6 s^{-1}, at least 2 orders of magnitude greater than k_d. Thus, the value of $R_{^3DOM^*}$ is approximately equal to R_{TMP}. Linear plots to determine k_{TMP} are presented in Figure S2,a.

FFA (50 μM initial concentration) was employed to measure the formation rate of $^1O_2$ ($R_{^1O_2}$, $M\ s^{-1}$), which could be determined by the following Equations [1,2]:

$$k_{FFA} = k_{FFA,^1O_2} \frac{R_{^1O_2}}{k_{FFA,^1O_2}[FFA]_0 + k_{^1O_2}} \quad \text{(S2)}$$

where k_{FFA} (s^{-1}) the pseudo-first order rate constant of FFA, $k_{FFA,^1O_2}$ is the second-order rate constant between $^1O_2$ and FFA (1.0×10^8 M^{-1} s^{-1}),[1] $k_{^1O_2}$ presents the physical quenching of $^1O_2$ by water (2.5×10^5 s^{-1}). Linear plots to determine k_{FFA} are presented in Figure S2,b.

The formation rate of $^•OH$ ($R_{•OH}$, M s^{-1}) were quantified by the addition of TPA (1 mM, non-fluorescent) and the detection of 2-hydroxyterephthalate (HTPA, fluorescent), which is the single hydroxylation product of TPA. This reaction has been shown to proceed with a yield of 35%. The generation of HTPA was linear with time (Figure S2,c). $R_{•OH}$ were calculated using the following equation [1]:

$$\frac{d[HTPA]}{dt} = 0.35 \times k_{\cdot OH,TPA} \times [TPA][\cdot OH]_{ss} = 0.35 \times R_{\cdot OH} \quad \text{(S3)}$$

where $k_{•OH,TPA}$ is the second-order rate constant between $^•OH$ and TPA (3.3×10^9 M^{-1} s^{-1}).

The quantum yields of $^3DOM^*$, $^1O_2$, and $^•OH$ were calculated using Equation 4 [1].

$$\phi_i = \frac{R_i}{I_0 \sum_{\lambda} F_{\lambda}(1 - 10^{\varepsilon_{\lambda} bc})} \quad \text{(S4)}$$

where R_i is the formation rate of $^3DOM^*$, $^1O_2$, and $^•OH$ in the HA fraction solutions, respectively, I_0 (Es L^{-1} s^{-1}) is the incident light intensity obtained by the *p*-nitroanisole/pyridine actinometer, F_λ is the spectral distribution of the light emitted by the lamp, ε_λ (L mg^{-1} cm^{-1}) is the absorption coefficient of DOM at a specific wavelength, *b* (cm) is the path lengthinside the reactor, and c (mg L^{-1}) is the concentration of DOM.

The solution screening factor (S_λ) is determined from solution absorbance (α_λ, cm^{-1}) and the light path length (*l*, cm) [3], as in Equation S5.

$$S_{\lambda} = \frac{1 - 10^{-\ell \times \alpha_{\lambda}}}{2.303 \times \ell \times \alpha_{\lambda}} \quad \text{(S5)}$$

Solution rates of light absorbance (R_{abs}) were determined from the photon flux (I_λ, Es cm^{-2} s^{-1}), measured solution absorbance (α_λ, cm^{-1}), and the screening factor (S_λ) [4], as in Equation S6.

$$R_{abs} = 2.303 \sum_{\lambda} I_{\lambda} \alpha_{\lambda} S_{\lambda} \quad \text{(S6)}$$

The photon flux (I_λ, Es cm^{-2} s^{-1}) is determined by PNA-pyridine actinometry. I_λ is determined as a function of $R_{a,PNA}$, S_λ, the molar absorptivity of PNA (ε_λ, M^{-1} cm^{-1}), and *l* as in Equation S7 [3]:

$$I_{\lambda} = R_{a,PNA} \sum_{\lambda} \frac{1}{2.303 \times S_{\lambda} \times \varepsilon_{\lambda} \times \ell} \quad \text{(S7)}$$

$R_{a,PNA}$ of an actinometer solution is determined from the pseudo-first-order loss rate of PNA ($k_{obs,PNA}$, s^{-1}), the initial concentration of PNA ($[PNA]_{t=0}$, M), and the quantum yield of reaction between PNA and pyridine (Φ_{PNA}) [3], as in Equation S8:

$$R_{a,PNA} = \frac{k_{obs,PNA}\left[PNA\right]_{t=0}}{\phi_{PNA}} \tag{S8}$$

Text S2

Analysis of Bromophenol Products, TMP, FFA, HTPA and PNA

A sample solution (20 mL) after irradiation was spiked with 1 μL 2-hydroxy-5-chlorobiphenyl (100 μg/L) as the internal standard, and then was acidified to pH ~2 using 2.5 M H_2SO_4, followed by extraction with dichloromethane (10 mL, 2x). After dehydration using an anhydrous sodium sulfate column, the extract was concentrated to 200 μL for direct analysis by GC–MS. Analysis was performed using an Agilent 7890 GC and 5975C MSD with an EI source operating in SIM mode, using ions at mass/charge ratios (m/z) 172 and 174 for bromophenols determination and 204 and 206 for 2-hydroxy-5-chlorobiphenyl determination. The GC was operated with helium as the carrier gas in the splitless mode with a DB-5ms capillary column (30 m × 0.25 mm × 0.25 μm). The following conditions were used for the analysis: a 1-μL injection, a source temperature of 230°C and an inlet temperature of 270°C. The column temperature ramp was as follows: 80°C for 2.0 min, 80–270°C at 15°C/min and 270°C hold for 10 min.

The concentrations of TMP, FFA and PNA were measured using a high-performance liquid chromatography (HPLC) system (Waters 717 plus) with a C18 column (5 μm, 250 × 4.6 mm, Agilent). The mobile phases were 70% acetonitrile/30% water with 10 mM phosphate buffer for TMP, 30% methyl alcohol/70% water for FFA, and 60% acetonitrile/40% water for PNA. The detection wavelengths were 280, 220 and 315 nm for TMP, FFA, and PNA, respectively. The concentration of HTPA was detected using a fluorescence spectrophotometer (λ_{ex} = 315 nm and λ_{em} = 425 nm).

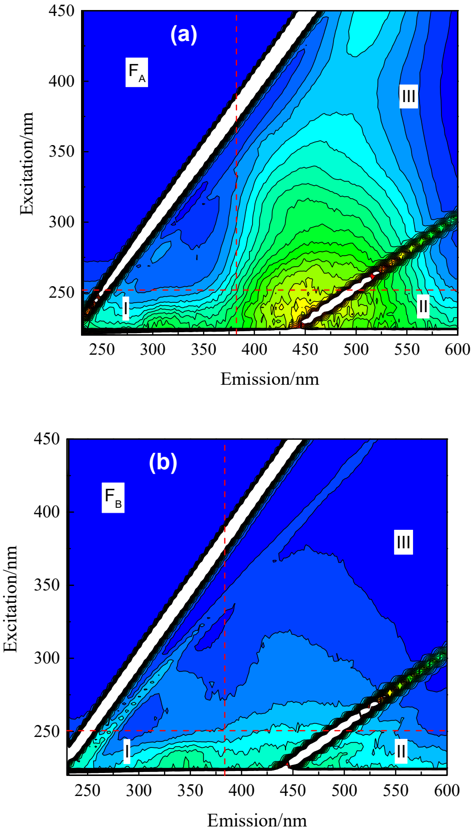

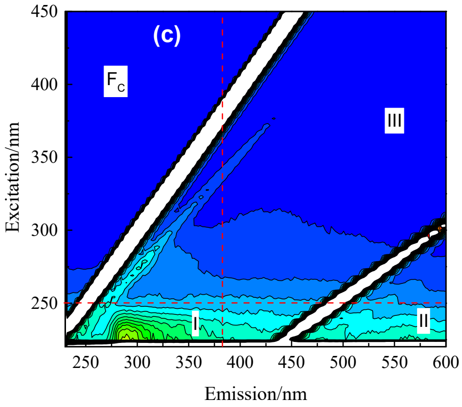

Figure S1 EEM fluorescence spectrum of F_A (**a**), F_B (**b**), and F_C (**c**).

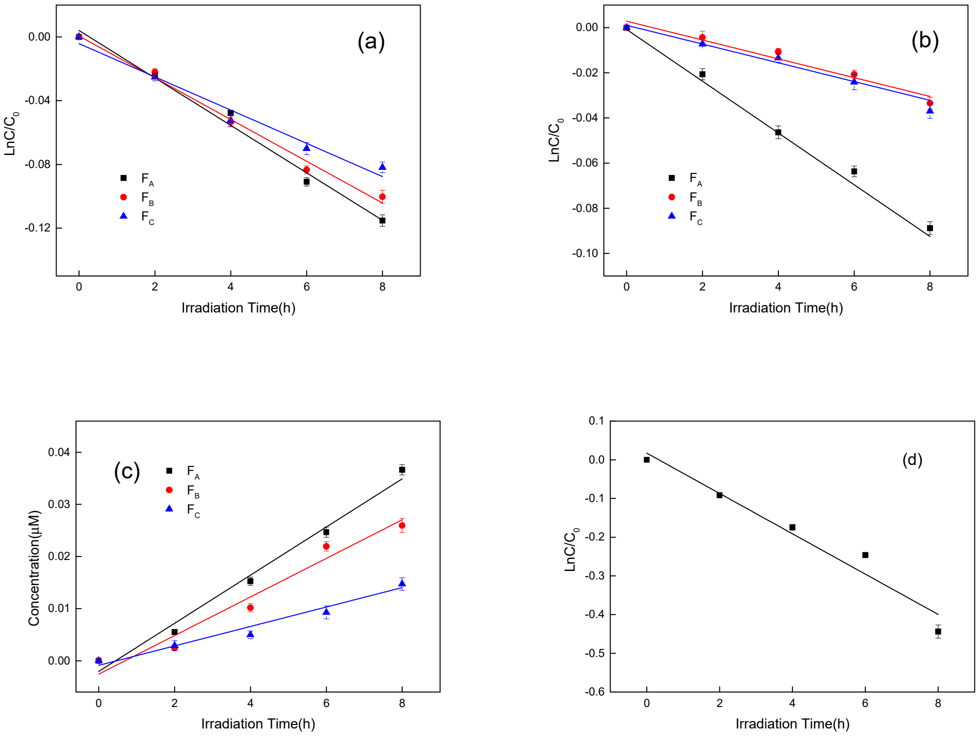

Figure S2 The curve of each probe molecule and PNA over time under simulated sunlight: (**a**) TMP, $[TMP]_0$=1mM; (**b**) FFA, $[FFA]_0$=50μM; (**c**) HTPA, $[TPA]_0$=1mM; (**d**) PNA, $[PNA]_0$=10μM.

References

1. Wan, D.; Sharma, V.K.; Liu, L.; Zuo, Y.; Chen, Y. Mechanistic Insight into the Effect of Metal Ions on Photogeneration of Reactive Species from Dissolved Organic Matter. *Environ. Sci. Technol.* **2019**, *53*, 5778–5786.
2. Marchisio, A.; Minella, M.; Maurino, V.; Minero, C.; Vione, D. Photogeneration of Reactive Transient Species upon Irradiation of Natural Water Samples: Formation Quantum Yields in Different Spectral Intervals, and Implications for the Photochemistry of Surface Waters. *Water Res.* **2015**, *73*, 145–156.
3. Maizel, A.C.; Remucal, C.K. Molecular composition and photochemical reactivity of size-fractionated dissolved organic matter. *Environ. Sci. Technol.* **2017**, *51*, 2113–2123.

4. Maizel, A.C.; Li,J.; Remucal, C.K. Relationships between dissolved organic matter composition and photochemistry in lakes of diverse trophic status. *Environ. Sci. Technol.* **2017**, *51*, 9624–9632.
